# Collective movement: reimagining children's physical literacy through shared responsibility

**DOI:** 10.3389/fspor.2026.1922306

**Published:** 2026-10-20

**Authors:** N. E. Houser, M. L. Humbert, L. J. Ferguson, L. M. Hellsten, A. L. Stoddart, M. C. Erlandson

**Affiliations:** 1 College of Kinesiology, University of Saskatchewan, Saskatoon, SK, Canada; 2 Faculty of Education, University of Winnipeg, Winnipeg, MB, Canada; 3 Faculty of Education, University of Regina, Regina, SK, Canada

**Keywords:** community, home, intervention, multi-context, multi-sectoral, school

## Abstract

**Background:**

Physical literacy, a multidimensional construct encompassing motivation, confidence, physical competence, and knowledge, is increasingly recognized as a critical determinant of lifelong movement. As a holistic construct, there is an opportunity to collaborative provide opportunities for physical literacy development in the many contexts that children move. As such, the purpose of this study was to examine the impact of a six-month multi-sectoral, multi-contextual physical literacy intervention implemented across home, school, and community contexts using a shared responsibility approach.

**Methods:**

A pre–post quasi-experimental design was employed with 686 children (mean age 10.5 ± 1.35 years) from intervention (*n* = 397) and comparison (*n* = 289) communities in Saskatchewan, alongside parent (*n* = 429) and teacher participants (*n* = 53). Physical literacy was assessed using the Physical Literacy Assessment for Youth (PLAY) tools, including measures of motor competence (PLAY fun), self-perception (PLAY self), parent perception (PLAY parent), and teacher perception (PLAY coach). A repeated measures mixed model was conducted as the primary analysis, with significance set at *p* < 0.05.

**Results:**

Results indicated a significant intervention effect for motor competence (*p* < 0.001), with the intervention group demonstrating significant improvements while the motor competence of the comparison group declined over time. Increases were observed across multiple domains, including locomotor skills, balance, and movement vocabulary. No significant group by time effects were observed for parental perceptions of children's physical literacy (*p* = 0.173), however there was a time effect which warrants further exploration (*p* < 0.001). No significant intervention effects were observed in children's self-perceived physical literacy (*p* = 0.784) or teacher-reported outcomes between groups (*p* = 0.987).

**Conclusion:**

These findings highlight the potential of a collaborative, community-wide approach to enhance key components of physical literacy, particularly motor competence and parent perceptions. The lack of change in self-perception measures suggests a need for stronger emphasis on psychological and affective domains within future intervention design. Overall, this study provides novel evidence supporting multi-sectoral, multi-contextual strategies as a promising pathway for fostering physical literacy development in children and underscores the importance of coordinated efforts across home, school, and community contexts.

## 1 Introduction

Physical literacy is defined as the “motivation, confidence, physical competence, knowledge and understanding to value and take responsibility for engagement in physical activities for life” (1, 2). Although the concept of physical literacy can be traced back to the late 1800s (3), in the past several years, physical literacy research has advanced significantly by adopting and creating principles and practices that can be applied across multiple sectors including education, sport, recreation, and public health (4–6). While the holistic nature of physical literacy [emphasizing physical, social, psychological and creative competencies (7)] makes it an encouraging approach to support movement opportunities throughout the life course, there can be challenges when trying to incorporate all (or multiple) domains of physical literacy effectively. Despite this challenge, incorporating multiple components of physical literacy is critical to its holistic development, and a sole focus on movement competence should be avoided when implementing physical literacy enriched opportunities (8).

Providing diverse movement experiences across environments (e.g., land, ice, snow, and water) and contexts (e.g., home, school, and community) is an essential tenet of physical literacy (9). One way to encourage movement opportunities in a variety of contexts is by involving multiple sectors in the implementation of physical literacy enriched initiatives (10). A physical literacy enriched initiative can be described as one that features opportunities to develop all (or many) of the components of physical literacy through the inclusion of pedagogically sound practices, developmentally appropriate activities, appropriate levels of challenges, and opportunities for progressions (4, 5, 9, 11). Unlike what is known about working in physical activity contexts, currently there is limited evidence on the impact of a multi-sectoral (e.g., sport, recreation, and education) shared responsibility approach to enriching the development of physical literacy in multiple contexts (e.g., home, school, and community). However, existing physical activity research may help in understanding how some individual components of physical literacy are influenced by different contexts and environments. For this study, a shared responsibility approach is one that emphasizes collective responsibility shared across individuals, families, communities, organizations, and governments, rather than relying on an individual group.

The environments and contexts in which children interact with daily include factors that present both facilitators and barriers to movement and physical literacy development. For example, aspects of the community including urban design, transportation, and park availability can influence physical activity levels (12, 13). More specifically, strategies such as supporting community physical activity opportunities, community planning and design, and physical activity outreach programs have proven to be positive intervention approaches for increasing physical activity levels (14). Alternatively, some community level barriers to physical activity for children may include limited recreation resources/availability, safety, and distance to facilities/resources (15). Similarly in schools, opportunities to influence physical activity exist as this is the context in which children may have the most movement opportunities, as the majority of children attend school (16), but schools cannot bear the sole responsibility. Barriers within the school context may include lack of opportunities for professional development, access to resources/space, and time (17). At home, opportunities for physical activity engagement allow for involvement of families, which is known to be a critical piece to children's movement behaviours (18), but limitations may surface around the valuation of movement in this context. For example, if parents do not value physical activity, children and their families are less likely to participate, whereas physical activity participation is higher when supported by parents (18). Across interventions that targeted multiple contexts, impacts were positive but uneven and usually specific to the outcome or context targeted. For example, one study combined classroom curricula and school-environment changes with parent classes, parent-child home assignments, household themed weeks, walk-to-school activities, and district competitions, increasing weekly PA (19). Another study used trained after-school staff, daily activity and nutrition opportunities, parent-child home-environment contracts, and Cooperative Extension coordination and observed increased after-school MVPA (20). Lastly, a movement-competence focused study combined home equipment and parent-led activity cards, facilitator-led school lessons with teacher development, and family activity nights and reported increased motor competence (21). Based on evidence to date, we can suggest that multi-context interventions can produce useful, context-specific gains, but success depends on what each context actually does, how well the components are coordinated, and whether the intervention reaches activity opportunities beyond the school day, so further investigation is needed.

In physical literacy enriched environments, similar features may be observed including positive physical activity experiences, peer and family support, and valuing of physical activity (22). From a physical literacy perspective, this multi-contextual approach may also support a shared responsibility for the development, implementation, and evaluation of interventions, where the onus to enhance physical literacy does not just lie with one group (e.g., parents or teachers), but as a collaborative effort of all members of a community. As demonstrated in our earlier research, a multi-contextual approach offers a promising outlet to positively influence children's physical literacy through collaboration within the community . Specific features of each context play an influential role on children's physical activity and physical literacy, and determining the right combination is integral to future physical literacy development and lifelong physical activity participation. As suggested, a multi-contextual (home, school, and community) intervention can offer many opportunities for enriching physical literacy, and one possible way to achieve this is through engaging multiple sectors (e.g., sport, recreation, and education) in intervention development and delivery. This collaborative approach may allow each sector to draw on their strengths and potentially fill gaps in the work of other sectors.

A multi-sectoral and multi-contextual approach holds significant promise for enhanced physical literacy opportunities, as this would support collaborative efforts that can successfully impact long term movement behaviours (23). Although there is existing research on physical literacy interventions (24), minimal evidence exists on the outcomes of a multi-sectoral intervention involving a combination of home, school, and community contexts. As such, the purpose of this study was to examine the impact of a six-month multi-sectoral, multi-contextual physical literacy intervention implemented across home, school, and community contexts using a shared responsibility approach.

## 2 Methods

Using a pre-post quasi-experimental design, the following measures were obtained at baseline and at six months post-intervention: the Physical Literacy Assessment for Youth (PLAY) tools (25); PLAY fun, PLAY self, PLAY parent, and PLAY coach. Ethical approval was granted from the  Behavioural Research Ethics Board, and divisional approval was granted from the partnering school division. Parents/guardians of participating children provided written informed consent and written child assent was obtained at the time of baseline data collection. Teachers and parents also provided written informed consent for completion of their respective questionnaires.

### 2.1 Participants

Students in grades 4 through 8 from three communities located in one school division (unit responsible for managing schools within a specific geographical area) in the province of Saskatchewan were invited to participate in this study, alongside their parents/guardians and physical education teachers. One community was designated as the intervention site (three participating schools), and two communities were designated as comparison sites (four participating schools), for a total of seven participating schools. The intervention community was selected by Saskatchewan in Motion (SIM), and comparison communities were selected based on similar population, available services, and socio-economic status to the intervention community.

### 2.2 Intervention

The intervention followed SIM five-step process as a framework for physical activity related community work (26). The five steps included: 1) Development of a community profile, 2) Engaging with the community, 3) Creating a physical literacy enriched community action plan, 4) Implementation, and 5) Evaluation. Step 1 involved obtaining a better understanding of the community through gathering information from surveys (including demographics) and focus groups (with students, parents, community officials, etc.). Step 2 was to share these findings with community members (e.g., parents, teachers, council members, and students) in a symposium. The findings from the community profile then led to Step 3 which was the formation of the action plan, done in collaboration with SIM, the Partners for Physical Literacy, and the community. Step 4 constituted the implementation of the action plan, where the community was mobilized to make changes and the intervention was delivered. Lastly, Step 5 focused on evaluation which allowed the Partners for Physical Literacy to determine the impact of the intervention, primarily done through data collection at the schools. Results presented are based on a culmination of all steps, but a focus in this study is placed on Steps 4 and 5. Activities included as part of the intervention (Step 4) took place between October and June (one school year) and are outlined below. It is important to note that intervention approaches were guided and co-created between the researchers and community, based on their expressed needs and values. The specific intervention strategies are detailed below and summarized in the Supplementary Table 1.

*Home:* In the home context, families received newsletters and infographics as part of the physical literacy awareness campaign in the fall; this was in addition to the consent forms and information pages all parents received. The awareness campaign provided an opportunity for any community members to get an understanding of the project and the concept of physical literacy, and can be seen as a strategy that bridged the three contexts (home, school, community). In the winter, the research team presented the physical literacy information at a School Community Council meeting to provide an understanding of the community's starting point. The Council consisted of parent representatives, school administrators, and school division leaders. The School Community Council presentation included a physical literacy education piece (e.g., what is physical literacy, why is it important, and how can it be developed), along with the presentation of physical literacy baseline data from the children in their community. A similar presentation was offered to all three schools, and one school accepted the presentation offer; this will be further discussed below. Families also had access to many of the intervention strategies delivered at the school and within the community, as any child-centered strategy typically includes some information for parents.

*School:* Prior to the initiation of any intervention activities in the schools, meetings occurred with administrative teams (principals and vice principals) to determine what each school needed to help them develop physical literacy in their students. The main need expressed by the administrative teams was support in physical education (e.g., physical literacy enriched physical education to meet curricular needs) as this school division did not have physical education specialists. In the school context, intervention strategies began as soon as the baseline data collection was completed. Throughout the intervention, regular meetings between the research team, school administrators and teachers were held to ensure the approach was in alignment with their needs as a school. As part of the awareness campaign in the fall, teachers were provided information and resources (e.g., websites) related to developing physical literacy. After baseline data collection, physical education curricular based physical literacy enriched lesson plans (20 per grade) were provided to all intervention schools along with the opportunity for a professional development session. In two of the three schools, professional development sessions were provided by the creator of the lesson plans (an experienced physical educator), to explain implementation and answer any questions the teachers had (the third school declined this opportunity). In the winter, the baseline data were presented to teachers at one school, while baseline results were shared with the other two schools based on their preference via the administrators in a Word document format. Additionally, in the winter, intervention schools were offered the opportunity to attend (at no cost) the annual Saskatchewan Physical Education Association Conference, two of the three schools involved in the intervention sent teachers to this conference. Lastly, physical literacy focused sport resources (e.g., sport specific skill development games/strategies) from several provincial sport governing bodies were delivered to all intervention schools, providing a previously underutilized opportunity to have sport organizations come into the school and teach their sport. Upon completion of the post-intervention data collection, a “thank you” was offered to participating students in the form of a “try-it” day. The “try-it” day involved participation from several sport organizations (e.g., golf, rugby, tennis, etc.) and the university, and provided opportunities for all students in grades 4–8 in the three intervention schools to participate in different sports. Teachers were in regular contact with the researchers throughout the academic year as they delivered the lesson plans, with any questions or clarifications, which also allowed the researchers to confirm delivery of the lessons.

*Community:* In the community context, a symposium in the fall, with students, teachers, parents, and community representatives (e.g., council members) in attendance, offered the opportunity to share the community profile, followed by idea generation sessions. The symposium allowed for the community itself to identify key action items and to form action teams to implement the items. The symposium was one of the first opportunities for any interested community members to become engaged in the process, and for Partners for Physical Literacy to gain an understanding of community needs. Community intervention strategies began with physical literacy information delivered through an awareness campaign in fall, which included a community webpage and newsletters that outlined physical literacy centered activities taking place in the community. In alignment with the campaign in the fall was the development of a communication plan to effectively circulate information about physical literacy events; this information was shared via avenues such as the local newspaper, in the mail, and online. At one of the large community events (“Activity Day”) in the fall, several provincial sport governing bodies were in attendance with opportunities for attendees to participate in different sports and learn how to become involved in the future. Lastly, in the winter, the community recreation plan was revamped to include more physical literacy enriched opportunities in their offerings. This recreation plan revamp made intentional connections between physical literacy and the different recreation opportunities available in the community, supporting increased awareness and knowledge about opportunities that support physical literacy development.

### 2.3 Procedure

#### 2.3.1 Data collection and assessors

The same group of assessors were used, and the same procedures followed for the baseline and follow-up data collection periods. Prior to the data collection, all assessors participated in a four-hour training session to learn how to assess participants using the PLAY fun tool. Training was conducted by a leader with extensive expertise in the PLAY fun tool, and followed procedures as outlined in the PLAY fun workbook (25). The research team was also trained to deliver the questionnaire (PLAY self, but this was typically done by one or two of the same researchers each time, while others assisted in checking the questionnaires over to ensure all questions were answered. Accessors were blinded to baseline values and grouping. Additionally, children were not able to watch their peers performing the task, limiting any potential observational learning or mimicking from occurring.

#### 2.3.2 Physical literacy measures

The PLAY tools were used to assess physical literacy in participating children (25). The PLAY battery of tools consists of PLAY fun: an 18-task measurement tool to determine children's motor competence; PLAY self: child evaluation of their self-perceived physical literacy; PLAY parent: parent's perception of their child's physical literacy; and PLAY coach: coaches, or for the purpose of this study, teacher's perceptions of a child's physical literacy level (25). The PLAY tools are one of the only battery of tools that offers an opportunity to gather information on multiple perspectives of physical literacy (e.g., child, parent, teacher, and researcher), which was critical for a more holistic picture of this multi-sectoral intervention.

Using PLAY fun, motor competence was scored on a visual analog scale of 0–100 (25). A total PLAY fun average motor competence score was determined by adding the scores of all 18 skills together, then dividing by 18. Average scores were also calculated for the motor competence sub-categories: locomotor; object control; and balance/stability. Movement vocabulary was determined from the PLAY fun scores based on the number of skills a participant scored over 50 on, as any skill with a score higher than 50 was considered as acquired. A PLAY fun construct validation study indicated an interrater agreement of ICC = 0.87 and a model fit of RMSEA = 0.055; CFI = 0.95; TLI = 0.94 (10). For our data specifically, internal consistency (as measured by Cronbach's alpha) was classified as good at both baseline and follow-up (*α* = 0.78 and *α* = 0.83).

The PLAY self questionnaire was administered by the assessors to measure participant's self-perceived physical literacy (27). Two sections of the PLAY self survey include environmental participation (6 items) and self-description of physical literacy (12 items). For this analysis, the PLAY self physical literacy score was determined based on the sum of the environmental participation and self-description sections, as done in Kriellaars et al. (2019) with the highest possible score of 78 (28). PLAY self validity studies report an internal reliability of Pearson Separation Index = 0.70–0.82 and a test-retest reliability of ICC = 0.81–0.84 and item and person fit residuals = −0.17–1.47 (27). For our data specifically, internal consistency for PLAY self (as indicated by Cronbach's alpha) was classified as good at both baseline and follow-up (*α* = 0.85 and *α* = 0.88).

The PLAY parent is a survey completed by one parent/guardian of each participating child, to assess parental perceptions of their child's physical literacy. The PLAY parent survey was sent home with participants after data collection at the school. PLAY parent is a 20-question survey with sections assessing parental perceptions around their child's cognition, motor competence, and environment. A total PLAY parent score is represented by the sum of questions 1 through 19 (question 20 is not included in the final score calculation), with a maximum possible score of 57. To date, no validity and reliability data on PLAY parent have been published. For our data specifically, internal consistency (as calculated by Cronbach's alpha) was classified as excellent for PLAY parent (*α* = 0.90 and *α* = 0.89), at baseline and follow-up respectively.

PLAY coach is a survey completed by coaches (teachers in this case), measuring their perceptions of the physical literacy of each participating child in their class. The PLAY coach tool has been used by teachers in previous studies in the school setting (29), and was appropriate for use in our study in its original form. PLAY coach is a 17-question survey featuring cognitive, environment, and motor competence sections. The survey was scored by calculating a sum of questions 1 through 16 (question 17 is not included in the final score calculation), with a maximum possible score of 80. To date, no validity and reliability data on PLAY coach have been published. For our data specifically, internal consistency (as calculated by Cronbach's alpha) was classified as excellent for PLAY coach (*α* = 0.99 and *α* = 0.98), at baseline and follow-up respectively.

### 2.4 Data cleaning and analysis

Prior to analysis and in addition to checking the overall dataset for outliers, 10% of data were checked for entry errors (e.g., data entry mistake where a “4” was entered instead of a “5” in a scale of 1–5) (30). A total of 26 errors were found and corrected out of a total of approximately 8,500 data points checked, which is a 0.3% error rate.

Descriptive statistics were computed to describe the sample, including demographic details such as age (mean ± standard deviation) and number of males and females. Additionally, spearman correlation analysis was conducted to determine the relationship between the physical literacy tools used. To analyze changes in physical literacy outcomes from baseline to endpoint, repeated measures mixed models were conducted (31). Separate models were fit for each of the physical literacy tools: PLAY fun- average motor competence; PLAY self- environmental participation and self-perceived physical literacy; PLAY parent- parent perceptions of child's physical literacy; and PLAY coach- teacher perceptions of child's physical literacy. The models included treatment group (intervention vs. comparison), time (baseline vs. endpoint), and group × time interactions as fixed categorical factors, while school was included as a random effect. To account for the dependency of repeated assessments within individual participants, participant ID was specified as a subject variable using an unstructured covariance matrix, while sex and grade were included as covariates. All analyses were performed using SPSS version 31 and significance was set at *p* < 0.05.

## 3 Results

### 3.1 Descriptives

A total of 686 children (344 female; 342 male) from the intervention (*n* = 397) and comparison (*n* = 289) groups with an average age of 10.5 ± 1.35 years, along with 429 parents (258 intervention; 171 comparison), and 53 teachers (representing data from 571 children; 329 intervention and 242 comparison) participated in this study at both time points. Table 1 demonstrates the spearman correlation coefficients between the physical literacy tools at baseline. All tools were significantly correlated, ranging from *r_s_* = 0.27–0.41.

**Table 1:** Correlations between baseline measures of physical literacy.

Measures	1	2	3	4
1. PLAY fun	-			
2. PLAY self	0.27 *	-		
3. PLAY parent	0.38 *	0.41*	-	
4. PLAY coach	0.32 *	0.30*	0.41*	-

Spearman correlation; *p < 0.001, Physical Literacy Assessment for Youth (PLAY).

### 3.2 PLAY fun

Average motor competence, as measured by PLAY fun (*n* = 686; 397 intervention and 289 comparison), demonstrated a significant intervention effect (time by group), *F*(1, 629) = 238.91, *p* < 0.001, as presented in Table 2. The comparison group demonstrated a significant decrease in average motor competence from baseline to follow-up, while the intervention group demonstrated a significant increase in average motor competence. There was no significant school effect, *F*(1,700) = 0.455, *p* = 0.50. A significant grade interaction existed *F*(4, 722.75) = 3.65, *p* = 0.006, for average motor competence scores, with *post hoc* analyses identifying that the intervention group was higher than the comparison group in all grades, but no significant sex interaction existed *F*(1, 724.34) = 0.13, *p* = 0.723.

**Table 2:** Summary of baseline and follow-up mean (SD) between groups for all PLAY tool measures.

Measures	Group	Baseline	Follow-up
PLAY fun	Comparison	50.87 (9.57)	47.07 (10.70)
	Intervention	47.49 (10.60)	55.62 (10.40)^a^
PLAY self	Comparison	59.09 (8.86)	59.15 (9.51)
	Intervention	58.18 (8.71)	58.28 (8.62)
PLAY parent	Comparison	30.14 (6.47)	31.08 (6.13)
	Intervention	29.45 (6.30)	31.25 (5.82)^a^
PLAY coach	Comparison	45.4 (15.60)	48.30 (17.00)^a^
	Intervention	45.90 (16.20)	49.10 (18.70)^a^

a Significant difference between baseline and follow-up (*p* < 0.05); Physical Literacy Assessment for Youth (PLAY).

Overall, there were significantly greater improvements in the intervention group than the comparison group in locomotor skills (for grades 4, 5, 6, and 7), object control (for grade 5), balance (all grades), and skills acquired (for grades 4, 5, 6, and 7). When comparing sexes within each group, few differences emerged, but significant differences did exist between sexes (boys > girls) for locomotor skills in grades 6 and 8 in the intervention group, and in number of skills acquired for participants in grade 8 in the intervention group. Out of the 18 skills measured, the average number of skills scoring above 50 for the intervention group was 10 at baseline and 12 at follow-up, while the comparison group had an average of 11 at baseline and 9 at follow-up. See Table 3 for a more detailed breakdown of PLAY fun sub-categories.

**Table 3:** PLAY fun sub-categories and skills acquired for intervention and comparison groups expressed as mean differences as determined by absolute change.

Skill	Gender	Grade 4	Grade 5	Grade 6	Grade 7	Grade 8
Locomotor (I)	Female	13.6 (10.2)	12.6 (9.5)	17.4 (11.8)^b^	7.3 (8.4)	7.2 (11.6)^b^
	Male	14.9 (9.0)	13.1 (11.5)	11.2 (12.5)	6.4 (11.1)	−1.9 (12.0)
	**Total**	**14.2** **(9.6)**^a^	**12.8** **(10.3)**^a^	**14.4** **(12.5)**^a^	**6.8** **(10.1)**^a^	**2.0** **(12.6)**
Locomotor (C)	Female	−4.2 (7.7)	−2.2 (6.8)	−1.3 (10.5)	−1.3 (7.5)	−4.8 (12.0)
	Male	−1.3 (8.3)	−2.3 (9.2)	−1.8 (7.9)	−1.9 (8.6)	−4.4 (11.3)
	**Total**	**−2.9** (**8.1)**	**−2.2** (**8.3)**	**−1.6** (**9.1)**	**−1.6** (**7.6)**	**−4.6** (**11.2)**
Object Control (I)	Female	−4.7 (10.6)	−2.6 (13.3)	−4.4 (10.3)	2.1 (10.6)	2.9 (11.3)
	Male	−0.8 (13.6)	−0.5 (11.7)	−0.5 (13.0)	1.2 (10.7)	1.7 (10.0)
	**Total**	**−2.9** **(12.1)**	**−1.8** **(12.7)**^a^	**−2.5** **(11.8)**	**1.6** **(10.6)**	**2.2** **(10.5)**
Object Control (C)	Female	−4.1 (10.1)	−6.7 (8.8)	−1.1 (9.4)	−1.8 (8.5)	2.9 (12.2)
	Male	−3.1 (12.8)	−4.7 (12.4)	2.9 (9.7)	5.3 (7.9)	1.6 (6.5)
	**Total**	**−3.7 (11.4)**	**−5.5** **(11.0)**	**1.1** **(9.7)**	**1.0** **(8.8)**	**2.3** **(9.7)**
Balance (I)	Female	15.6 (10.4)	20.5 (15.5)	15.1 (14.6)	11.6 (13.8)	11.1 (14.3)
	Male	16.6 (13.2)	19.5 (17.5)	17.7 (15.6)	7.6 (12.3)	10.2 (14.9)
	**Total**	**16.0** **(11.7)**^a^	**20.1** **(16.3)**^a^	**16.4** **(15.1)**^a^	**9.2** **(13.0)**^a^	**10.6** **(14.5)**^a^
Balance (C)	Female	−9.8 (10.4)	−10.1 (10.6)	−1.6 (11.5)	−4.8 (7.1)	−9.7 (6.6)
	Male	−7.8 (12.1)	7.1 (9.5)	−4.8 (11.5)	−8.0 (7.7)	−2.6 (17.3)
	**Total**	**−8.9** **(11.2)**	**−8.4** **(10.1)**	**−3.4** **(11.5)**	**−6.0** **(7.3)**	**−6.6** **(12.8)**
Skills Acquired (I)	Female	3.4 (2.4)	2.9 (2.1)	2.5 (2.7)	2.1 (2.5)	1.8 (3.3)^b^
	Male	3.2 (2.4)	3.2 (3.0)	2.1 (2.8)	1.6 (3.4)	−0.4 (2.1)
	**Total**	**3.3** **(2.4)**^a^	**3.0** **(2.5)**^a^	**2.3** **(2.7)**^a^	**1.8** **(3.0)**^a^	**0.5** **(2.9)**
Skills Acquired (C)	Female	−2.3 (2.6)	−2.2 (2.6)	−1.7 (2.5)	−0.4 (2.6)	−0.8 (1.9)
	Male	−1.0 (2.4)	−1.3 (2.5)	−1.5 (2.4)	0.3 (2.2)	−0.7 (2.6)
	**Total**	**−1.7** **(2.6)**	**−1.7** **(2.6)**	**−1.6** **(2.4)**	**−0.1** **(2.4)**	**−0.7** **(2.2)**

I, intervention; C, comparison.

Mean differences in motor competence as determined by absolute change.

Values expressed as mean (standard deviation).

a significant difference between comparison and intervention group (*p* < 0.05).

b significant difference between female and male within intervention or comparison group.

### 3.3 PLAY self

Analysis of the PLAY self (*n* = 686; 397 intervention and 289 comparison) environmental participation and self-description components demonstrated no significant intervention effect (time by group), *F*(1, 643) = 0.075, *p* = 0.784 (Table 2). Additionally, there were no significant grade, *F*(4, 717.81) = 0.92, *p* = 0.453, or sex, *F*(1, 791.69) = 0.94, *p* = 0.333, interactions for PLAY self scores. There was a significant school effect, *F*(1,772)= 13.63, *p* < 0.001.

### 3.4 PLAY parent

Parental perception (*n* = 429 parents; 258 intervention and 171 comparison) of their child's physical literacy **(**PLAY parent) demonstrated no significant differences in time by group, *F*(1, 586) = 1.87, *p* = 0.173, but there was a significant time effect, *F*(1, 586) = 19.44, *p* < 0.001 (Table 2), which upon further exploration is due to an increase in parental perceptions in the intervention group. There were no significant grade *F*(14, 643.89) = 0.45, *p* = 0.773 or sex, *F*(1, 667.88) = 0.00, *p* = 0.965 interactions for PLAY parent scores. Additionally, there was no school effect, *F*(1,764) = 2.92, *p* = 0.088.

### 3.5 PLAY coach

In a sample of 53 teachers (representing data from 571 children; 329 intervention and 242 comparison), no significant differences were found between the intervention and comparison groups (group by time), *F*(1, 608) < 0.001, *p* = 0.987, in PLAY coach, a measure of the teacher perceptions of the child's physical literacy (Table 2). There was a significant increase in PLAY coach scores for both groups from baseline to follow-up (time), *F*(1,607) = 25.93, *p* < 0.001 and a significant difference between groups at both timepoints (group), F(1,1217) = 15.87, *p* < 0.001. Additionally, there was no significant grade interaction, *F*(4, 355.95) = 1.73, *p* = 0.143, and no significant sex interaction, *F*(1, 340.91) = 0.22, *p* = 0.642 for PLAY coach scores. There was also a significant school effect, *F*(1,813) = 7.72, *p* = 0.006.

## 4 Discussion

To the authors' knowledge, this is one of few studies to explore the impact of a multi-sectoral (e.g., sport, recreation, and education) and multi-contextual (e.g., home, school, and community) physical literacy intervention. The six-month intervention resulted in significant intervention effects (group × time) on average motor competence (measured by PLAY fun), as well as significant time effects for both parent perspectives of their child's physical literacy (measured by PLAY parent) and teacher perspectives of their child's physical literacy (measured by PLAYcoach). The findings presenting a promising step to informing future practice and interventions, and suggest that a shared responsibility approach to enriching physical literacy can be impactful, but there is also a need for further exploration into ensuring holistic physical literacy development (of all components) and clear multi-sector contributions. In particular, this study identified that there is encouraging potential for physical literacy development when different sectors work together, as this can remove barriers that single groups often experience, and can help to fill some gaps that each organization might have (financial, time, personnel, or otherwise) <BLINDED REF>. Additionally, the correlation analysis identified that all tools were significantly correlated (low-moderate correlations), suggesting an alignment exists between self- and external perceptions of physical literacy.

Average motor competence of participants (PLAY fun) was significantly different between the intervention and comparison groups at both timepoints. Further, the comparison group observed a significant decrease in average motor competence between baseline and follow-up while the intervention group had a significant increase. The intervention group improved their average motor competence from 48 at baseline to 56 at follow-up (*d* = 1.09), a change that shifts participants from an emerging to a competent classification (10). This finding is even more encouraging given the potential for significant decreases in motor competence as observed in the comparison group, with a decrease from 51 to 47 (*d* = 0.66). In addition to average motor competence, movement vocabulary, as determined by the number of skills acquired, is also important to note. Out of the 18 skills measured in PLAY fun, the average number of skills scoring above 50 for the intervention group was 10 at baseline and 12 at follow-up (*d* = 0.90), while the comparison group had an average of 11 at baseline and 9 at follow-up (*d* = 0.62). The Saskatchewan physical education curriculum identifies a series of motor skills and patterns that should be acquired by the time students complete grade 5 (32). Given these curricular expectations, the 18 skills measured in PLAY fun should have been well understood and performed (i.e., acquired) by grade 5, but this was not the case in the current study. Although the increase in the intervention group movement vocabulary is encouraging, with the average age of this sample being 10.5 years (placing them in grade 5), students are not meeting the curricular expectations of possessing a range of well understood and performed skills by this age, and similar findings have been reported in other studies (33). A further exploration of the PLAY fun sub-components is necessary as differences were identified between groups in locomotor skills, object control, and balance. Based on our current understanding of the importance of a variety of movement experiences for well-rounded physical literacy development (8), the difference evident in the PLAY fun sub-components (i.e., locomotor, object manipulation, balance) may provide insight into the types of movement opportunities offered to children, and how these opportunities influence holistic physical literacy development.

Physical literacy environmental participation and self-description components of the PLAY self tool (25) revealed no significant difference between participants in the comparison or intervention group at either timepoint, and no significant change in either group between the baseline and follow-up measures. Although some physical literacy intervention studies have observed changes in environmental participation and self-description of physical literacy (PLAY self) (28), others have observed no changes in PLAY self scores (34), as was found in the current study. There are two factors that may have contributed to the lack of change in PLAY self scores. First, there is a possibility that the intervention targeted motor competence domains and neglected the motivation and confidence related components. As physical literacy is a holistic concept, physical competence and confidence should be developed concurrently (5, 35), but the implementation of this can be complex. The challenge around concurrently developing confidence and competence could partly be attributed to the intervention development, and a slight favouring of motor competence compared to confidence and motivation, as motor skill development is not always accompanied by strategies to intentionally enhance confidence and motivation. Additionally, the intervention delivery could have been partially influenced by the ability, confidence, comfort level, and knowledge base of teachers and parents, as familiarity with physical literacy ranged in this study, with some individuals hearing about it for the first time to others familiar for an extended period prior to this study. Future strategies should feature an enhanced emphasis on the other components of physical literacy (e.g., confidence, motivation, and active participation) which are often less intuitive to incorporate.

Emphasizing confidence, motivation, and active participation may be done through strategies that focus on strong pedagogical approaches and offering opportunities for positive challenge in a variety of different contexts (5). Creating an environment where children can comfortably explore movement and experience positive challenges is a pedagogical skill that takes more time and experience than teaching basic motor skills. Although the pedagogical factors in physical literacy were emphasized in the teacher lesson plans distributed to all teachers in the intervention schools (through encouraging all components of physical literacy), only some teachers attended the professional development for these plans and one school declined the professional development session, which may have limited the application of the suggested strategies. Second, some of the components measured in PLAY self such as confidence and motivation may be more stable, slower changing variables in comparison to variables like motor competence, as it has been suggested that cognitive abilities build on motor skill development (36). The current findings also note a possible discrepancy between average motor competence (PLAY fun) and the self-perceived physical literacy (PLAY self) score, where motor competence scores were typically lower than the child's self-perceived physical literacy, and this did not differ between sexes. Although these tools measure different things, it may represent a mismatch between actual and perceived abilities. This is congruent with current research that suggests there is a low association between actual and perceived motor competence, where perceptions are typically much higher than actual competence, but further research is warranted to better understand the impact of this relationship (37). This mismatch between actual and perceived abilities is not necessarily negative, as it may encourage greater participation if confidence is present, but again, further research must be conducted to gain a better understanding of this relationship.

Parent perspectives of their child's physical literacy, as measured by PLAY parent (25), demonstrated no intervention effect, but a significant time effect in the PLAY parent scores. This significant time effect warranted further exploration, which appears to demonstrate an increase from baseline to follow-up in the intervention group. Although no differences exist between the groups, the upward change in parent perceptions of their child's physical literacy in the intervention group represents a promising finding for this study warranting further exploration. In this study, as part of the “home” component of the intervention, parents were primarily provided with resources and educational tools to learn about physical literacy as part of the awareness campaign. In addition, parents may have been informed about physical literacy from their children and their experiences with the study (e.g., reading the consent forms and looking at the infographic). With these campaign components and their child's participation in the study, parents may have gained a better understanding of their child's abilities, while also observing positive changes in their child's motor competence. Parents play a critical role in physical activity and sedentary behaviours (38), and a similarly critical role of parents exists in physical literacy contexts (8); therefore, their perspectives of their children's abilities can contribute to movement related behaviours. Parent perspectives and actions, particularly related to parental self-efficacy, parenting styles, and perceived importance of physical activity can all play a positive role on their children's behaviours.

As influential role models in children's lives, it was important to understand the perspectives of teachers. Teachers' perceptions of their students' physical literacy, measured by PLAY coach (25), revealed no significant difference between groups at baseline or follow-up, but both groups reported a significant increase in PLAY coach scores over time. These findings suggest that the intervention had little effect on the perceptions teachers had of their students' physical literacy, but involvement in the study may have influenced perceptions, as both groups demonstrated a significant increase in PLAY coach scores between baseline and endpoint. It is worth noting that there were no physical education specialists in the elementary schools in this school division, and all physical education classes were therefore taught by classroom teachers. Although only the intervention group received the home, school and community interventions related to the study, involvement in a physical literacy study may have influenced teacher perceptions overall. Involvement in the study (in either group) meant teachers were exposed to physical literacy information through the PLAY coach assessment, which may have encouraged further emphasis or exploration into physical literacy or what features to look for in their students moving forward. There is also the possibility that the timing of measurements played a role in how well the teachers knew the children, as the baseline data collection was done at the start of the school year and follow-up measures occurred after teachers had worked with the students for six months. Additionally, development of the students' skills over time was based on how many times teachers had physical education classes to deliver movement opportunities to participating children (e.g., curriculum delivery, ranged from 2 to 4 times a week); therefore, resulting in a change in perception, but also a change in ability of all children from baseline to follow-up. Further, many of the intervention strategies took place in the school context, with some, but fewer occurring in the home and community contexts. Beyond the argument that schools can be opportune locations to reach many children in one context (23), there may exist other reasons for the uneven distribution of intervention strategies (e.g., expertise of the team delivering intervention) in the current study related to intervention development and delivery as well as roles of the partners delivering the program. This is further explored in previous research < BLINDED REF>. It is also important to note that this study included one intervention community and two comparison communities which is an important consideration as intervention conditions cannot be fully separated from community level characteristics, despite best efforts in matching these communities.

## 5 Conclusion

Overall, this study sought to determine the impact of a multi-sector intervention with multi-contextual initiatives taking place through a shared responsibility approach. This study demonstrated that although not all physical literacy components were positively impacted by the six-month intervention, children's motor competence had positive intervention effects, in addition to promising findings related to parental perceptions of their child. As one of the first of its kind to be documented, this multi-contextual and multi-sectoral intervention provides insight into strategies to enhance components of physical literacy in children at the home, school, and community levels, while also highlighting an opportunity for future programs to place more emphasis on the social and psychological components of movement and further explore the potential of a partnership approach. This study begins to suggest that a shared responsibility approach to physical literacy, as opposed to siloed approaches, has the potential to create positive and significant change. More research is needed to determine if this approach may have an overall impact on enriching physical literacy experiences, and further support downstream effects such as physical activity and health outcomes.

## Data Availability

The raw data supporting the conclusions of this article will be made available by the authors, without undue reservation.

## Supplementary material

Supplementary Table 1 Intervention actions in the home, school, and community contexts.
