# Supplementary material for: Collective movement: reimagining children's physical literacy through shared responsibility
Source: Front Sports Act Living. 2026 Oct 20;8:1922306. doi: 10.3389/fspor.2026.1922306 (PMC13727537; doi:10.3389/fspor.2026.1922306)
Supplement: Supplementary file 1 [file Table1.docx]

**Supplementary Table**

*Supplementary Table. Intervention actions in the home, school, and community contexts*

|  | **Context** | | |
| --- | --- | --- | --- |
|  | **Home** | **School** | **Community** |
| **Fall** | Infographic & newsletter inserts as part of the awareness campaign (sent home by schools) | Teacher specific information and resources (awareness campaign) | Community page & the awareness campaign (including inserts in newspapers/water bills) |
|  |  | Lesson plans, 20 per grade (and professional development for teachers) | Development of communication plan between SIM, community leaders, and community members |
|  |  |  | Symposium (for information sharing and idea generation) |
|  |  |  | Presentation to City Council regarding the project and their involvement |
| **Winter** | School Community Council meeting presentation (Overview of physical literacy and baseline data) | Complementary registration for the Saskatchewan Physical Education Association Conference offered to interested teachers at each school | Provincial Sport Governing Bodies attend community “Activity Day” event to present opportunities |
|  |  | Baseline data presentation to teachers (1 school) | Redesigned recreation programs to include physical literacy language/approaches |
|  |  | School sport resources distributed |  |

*Note: Resources developed by Saskatchewan in Motion can be found at: <https://www.in-motion.ca>
